# Planar cell polarity proteins determine basal cell height in the later stage embryonic mouse epidermis'

**DOI:** 10.12688/wellcomeopenres.17733.2

**Published:** 2023-01-19

**Authors:** Carl Hobbs, Caroline J. Formstone

**Affiliations:** 1 Wolfson CARD, King's College London, London, SE1 1UL, UK; 2 Department of Clinical, Pharmaceutical and Biological Sciences, University of Hertfordshire, Hatfield, AL10 9AB, UK; 3 Centre for Developmental Neurobiology, King's College London, London, SE1 1UL, UK

**Keywords:** mouse epidermis, PCP, cell shape, coordination across tissue layers

## Abstract

**Background:** Complex organ formation requires the coordinated morphogenesis of adjacent tissue layers. Here, we report a role for the planar cell polarity (PCP) proteins Fz6 and Celsr1 in generating squamous basal cells in the later stage embryonic epidermis of the mouse is reported, which may impact upon the shape of overlying suprabasal cells.

**Methods: **The depth of the epidermis and basal layer as well as cell proliferation index was scored from immunostained wax sections taken from different mouse embryos mutant in planar cell polarity signalling and their wild-type littermates. Orientation of epidermal cell division in *Celsr1* *Crash/Crash* mutants was determined from thick frozen immunostained sections. Immunostained wax sections of wild-type skin explants cultured using the Lumox method enabled any changes in epidermal and basal layer depth to be measured following the release of surface tension upon dissection of skin away from the whole embryo.
**  **

**Results:** Increased numbers of columnar and cuboidal basal epidermal cells were observed in *fz6-/- *mutant and *Celsr1* mouse mutant *Crash/Crash* which correlated with visibly more rounded suprabasal cells and a thicker epidermis.

**Conclusions:** Altogether these data support tissue intrinsic roles for PCP proteins in ‘outside-in’ (radial) skin architecture.

## Introduction

Complex organs comprise multiple tissue layers. During organ formation, morphogenesis of the different tissue layers is coordinated to achieve the correct architecture for healthy organ function. A useful model for this is the mammalian embryonic epidermis which is a stratified epithelium comprising of a progenitor basal cell layer, a suprabasal layer and a surface periderm layer ( Hu *et al.*, 2018). Recent studies have revealed two independent phases of suprabasal layer formation. A nascent suprabasal layer emerges via delamination of basal progenitors and is proliferative ( Damen *et al.*, 2021; Miroshnikova *et al.*, 2018; Williams *et al.*, 2014). Subsequently, the suprabasal layer differentiates and become highly keratinised. Later stage suprabasal cells (E15.5 onwards) are generated via asymmetric cell division of basal progenitors ( Damen *et al.*, 2021; Lechler & Fuchs, 2005; Williams *et al.*, 2014).

 In addition to epidermal stratification, embryonic skin becomes globally and locally patterned along the tissue plane. The core planar cell polarity (PCP) pathway plays a major role in this process which manifests in the orientation and alignment of hair follicle down-growth along the head-to-tail axis of mouse back skin ( Cetera *et al.*, 2018; Chang *et al.*, 2016; Devenport & Fuchs, 2008; Wang *et al.*, 2010). As hair placodes form, PCP proteins exhibit planar enrichment at opposing interfaces between basal progenitor cells along the head-to-tail axis ( Cetera *et al.*, 2018). Two seven-pass transmembrane receptors *frizzled6* ( *fz6*; Wang *et al.*, 2006) and *Celsr1* (homologue of Drosophila *flamingo*; Hadjantonakis *et al.*, 1998; Usui *et al.*, 1999) play important roles in planar hair follicle alignment as their loss of function ( *fz6-/-*, *Celsr1-/-*) leads to randomisation of hair follicle orientation in adult back skin ( Ravni *et al.*, 2009; Wang *et al.*, 2010). The same phenotype is reported for embryonic E16 and post-natal back skin of *fz6-/-* embryos ( Chang *et al.*, 2016; Wang *et al.*, 2010), indicating on-going roles for PCP proteins in global alignment of epidermal appendages. Notably, live imaging of E15.5 *fz6-/-* embryonic explants demonstrated loss of head-to-tail alignment of local cell rearrangement in hair placode cells ( Cetera *et al.*, 2018). Proposed dominant mutations in *Celsr1* ( *Crash;Crsh,* Curtin *et al.*, 2003) and in the four-pass transmembrane PCP protein *Vangl2* ( *Loop-tail:Lp*, Murdoch *et al.*, 2001) together with *Vangl1/Vangl2* double knockouts ( Cetera *et al.*, 2018) however, lead to hair follicle growth vertically downwards into the underlying dermis rather than at an oblique angle, as in wild-type ( Devenport & Fuchs, 2008), which implies that PCP proteins play a primary role at the local cell level. Live imaging of E15.5 *Vangl1/Vangl2* double knockouts reveals severe reduction/loss of hair placode cell rearrangements ( Cetera *et al.*, 2018). Recent study of *Crsh* and *Lp* homozygote mutants has uncovered epidermal defects along an ‘outside-in’ tissue axis ( Box *et al.*, 2019; Panousopoulou *et al.*, 2016), which can also be defined as ‘superficial-basal’ in terms of epidermal architecture or ‘radial’: effectively the ‘radial’ tissue axis is perpendicular to ‘planar’. The study of Panousopoulou *et al.* (2016) proposes that radial cell rearrangements within the surface ectoderm at E13.25 fail in *Celsr1 Crsh/Crsh* mutants. Here it is reported that *Crsh* homozygotes together with *frizzled-6* ( *fz6*) null mutants exhibit an abnormal thickening of the later stage epidermis (E17-E17.5), which correlates with a loss of squamous basal cells. Increased numbers of taller basal cells in each mutant correlate with more rounded suprabasal cells. Altogether the data presented is consistent with a model where PCP mouse mutants exhibit a late stage, tissue intrinsic failure to locally generate or maintain squamous basal cell shape which may directly impact on suprabasal cell shape. This is exciting because PCP proteins are expressed within the basal epidermal layer, raising interesting questions about how basal layer PCP signalling impacts upon adjacent, overlying suprabasal layers. These data also provide additional support for core-PCP protein function(s) in radial skin architecture.

## Methods

### Ethical statement

We expected mice litter sizes of 4–8 with at least one homozygote mutant per litter, thus we determined that for each condition six pregnant females would be required to guarantee sufficient control and mutant embryos for an experimental analysis with n=3 embryos. The same embryos were used for tissue height and proliferation analyses. Two fertile heterozygote males only for each genotype were maintained throughout the study. All mice were bred according to UK Home Office guidelines under Project Licence that was reviewed by the Animal Welfare and Ethical Review Body (AWERB) at King’s College London before Home Office review, the Establishment Licence number for King's College London is X24D82DFF. Prior to individual studies taking place, a study plan was also reviewed within King’s College London. Mice were held in individually ventilated cages at an average 21°C within a 12h light/dark cycle with food and water provided ad-lib. Mating trios (one male, two females) were set up and monitored morning and afternoon until all females had plugged. At desired gestational age, pregnant females were killed via schedule 1 (cervical dislocation) with all embryos killed by hypothermia and exsanguination.

### Mice

The *fz6* and *Celsr1* mouse mutants have been described previously ( Curtin *et al.*, 2003; *Crsh/Crsh*: Guo *et al.*, 2004; *fz6*-/-: Ravni *et al.*, 2009; *Celsr1-/-*) *.* Genotypes are: *Crsh/Crsh*, inbred BALB/c strain; *fz6*-/-, C57BL6 × 129 background; *Celsr1-/-*, floxed mice 129 x 1/Sv 129S1/Sv background ( http://www.informatics.jax.org/allele/key/606422) were crossed with the germline deleter PGK-Cre and the heterozygous progenies ( *Celsr1*+/-) were inter-crossed to generate homozygous mutants ( *Celsr1*-/-) and controls ( *Celsr1*+/+). All mice were between 8 weeks and 1 year of age. Mice were genotyped by polymerase chain reaction (PCR). Wild-type mice used for explant cultures were the outbred CD-1 strain (in-house). Timed mating was performed and 9am of the first day of plugging was taken as E0.5. Wild-type and homozygous mutant mice from the same litters were compared. No inclusion or exclusion criteria were set, no embryos were excluded from any of the analyses performed. Mutant embryos were not randomised but due to variability in *Celsr1-/-* embryo phenotypes data from 3 representative embryos are shown of n=8 *Celsr1-/-* embryos analysed. One wild-type embryo for each condition was randomly chosen for data analysis. Analyses were carried out as outlined in ARRIVE checklist ( Formstone, 2022).

Risk assessments were generated prior to experimentation.

### Histology and immunohistochemistry

*Paraffin sections* were generated and immunostained according to Oudin *et al.*, 2011. Haematoxylin and Eosin ( *H&E) staining* was as described in Panousopoulou *et al.* (2016). *Suspension explants* were embedded into wax as described in Panousopoulou *et al.* (2016). Wax sections were photographed using a Zeiss HBO50 light microscope with an Axiocam 503 colour camera and Axiovision rel.4.7 software (Zeiss). Images were exported into Adobe Photoshop CS5.1 and manipulated using the *crop* function.

The primary antibodies used were cytokeratin1 (K1) antibody (rabbit polyclonal, 1:500; Covance AF109), Ki67 (mouse monoclonal, 1:1000; BD Pharmingen, clone 56, 556003) and PhosphoHistoneH3 (PHH3; rabbit polyclonal. 1:300, Millipore 06-570). Secondary antibodies (Life Technologies) were fluorescently labelled and used at 1:1000.

### Measurement of total epidermal thickness and basal cell height

For whole embryos, epidermal thickness and basal cell height measurements were from longitudinal wax sections, every 10 basal cells across inter-follicular epidermis from mid-trunk back skin. Immunostained or H&E-stained wax sections of epidermis were viewed under 40X magnification, bright field illumination and measurements were taken using the *measure* tool of Axiovision rel.4.7 software (Zeiss). Measurements for total height were excluded if the epidermis in section appeared distorted. For each basal cell measured, total height of the overlying epidermis including the height of the basal cell was also scored. At least 75 measurements were made from three independent sections from each of n=3 embryos (from two litters) for each condition. Total adult animals used was n=6. Analysis was blinded, embryos embedded in wax for each gene knockout condition and wild-type were assigned a number by the histologist. One wild-type was randomly chosen for analysis from each mutant cohort. Mutant embryos (n=8) from more than two litters were analysed for *Celsr1-/-* to confirm the variability in phenotype observed in original litters for one embryo. Embryos exhibiting representative phenotypes are shown for *Celsr1-/-*. Immunostained sections were analysed using Axiovision rel 4.7 software, measurements were taken and then the genotype linked to each number was revealed. Histograms were generated using Graphpad Prism 7, but Microsoft Excel could also be used. Statistical analysis was one-way ANOVA with Bonferetti post-hoc test, using Prism software. Frequency graphs, correlation plots and their R-squared values were generated by Prism software.

For suspension explants, five measurements of epidermal thickness and basal cell height were taken from a wax section derived from the central portion of each explant, n=3 explants, total of 15 measurements, using the measure tool of Axiovision rel 4.7 software. For each basal cell measured, total height of the overlying epidermis including the height of the basal cell was again scored. A total or two measurements were taken from each mid-flank of n=3 transverse wax sections taken from each of n=3 mouse embryos, total of 18 measurements. All embryos were taken from the same two wild-type litters. Total adult animals used was n=2. Histograms were generated using Graphpad Prism 7. Statistical analysis was unpaired two-tailed Student’s t-test, using Prism software *.* Analysis of explants was not blinded.

### Determination of cell proliferation

Ki67 staining was used to assess any unusual patterns of cell proliferation. Mitotic index was scored manually using immunostaining for pHH3 and H&E staining of telophase divisions from three images analysed from three embryos (from two litters) for each condition, basal and suprabasal divisions were scored for each 1000 basal cells counted, n> 1000 basal cells for each condition. Total adult animals used was n=6, the same embryos were used to score for cell proliferation as were used to measure epidermal thickness. Histograms were generated using Graphpad Prism 7 and statistical analysis was unpaired two-tailed Student’s t-test, using Prism software. Analysis was blinded, embryos embedded in wax for each condition were assigned a number by the histologist. Immunostained sections were analysed using Axiovision rel 4.7 software, measurements were taken and then the genotype linked to each number was revealed.

### Quantification of oriented cell division

Thick longitudinal frozen sections (100μm) were immunostained with fibronectin to label the basal lamina, E-cadherin to label cell outlines and DAPI to label nuclei. Sequential images along a stretch of mid-trunk back skin were taken on a confocal microscope (Nikon A1R). Z-stack images were generated using 0.3µm steps. For each image, 3-D reconstructions of each Z-stack using Volocity software were generated (see Figure 2C). All telophase divisions within inter-follicular epidermis for which chromatids were fully visible within the Z-stack and their underlying basal lamina were individually cropped and the resulting images rotated (3D opacity mode) until the segregation of each chromatid pair could be measured relative to the basal lamina (marked by staining for fibronectin). One snapshot was taken from opposing sides of any given division. Angles of cell division relative to the basal lamina were measured using the *ruler* tool in Adobe Photoshop CS5.1. In each case, a line was first drawn along the basal lamina and then through the centre of each chromatid, which appeared in side-view. The mean angle for each division (of the two angles derived) was calculated. A total of n=37 wild-type divisions and n=36 *Crsh/Crsh* divisions were analysed from skin sections taken from n=3 embryos (from four litters) for each condition. Total adult animals used was n=4. Statistical analysis using unpaired two-tailed Student’s t-test compared wild-type and *Crash/Crash* divisions in bins shown, significant differences were not found. Histograms were generated using Graphpad Prism 7 and statistical analysis was unpaired two-tailed Student’s t-test, using Prism software *.* Analysis was not blinded.

## Results

**Figure 1.**
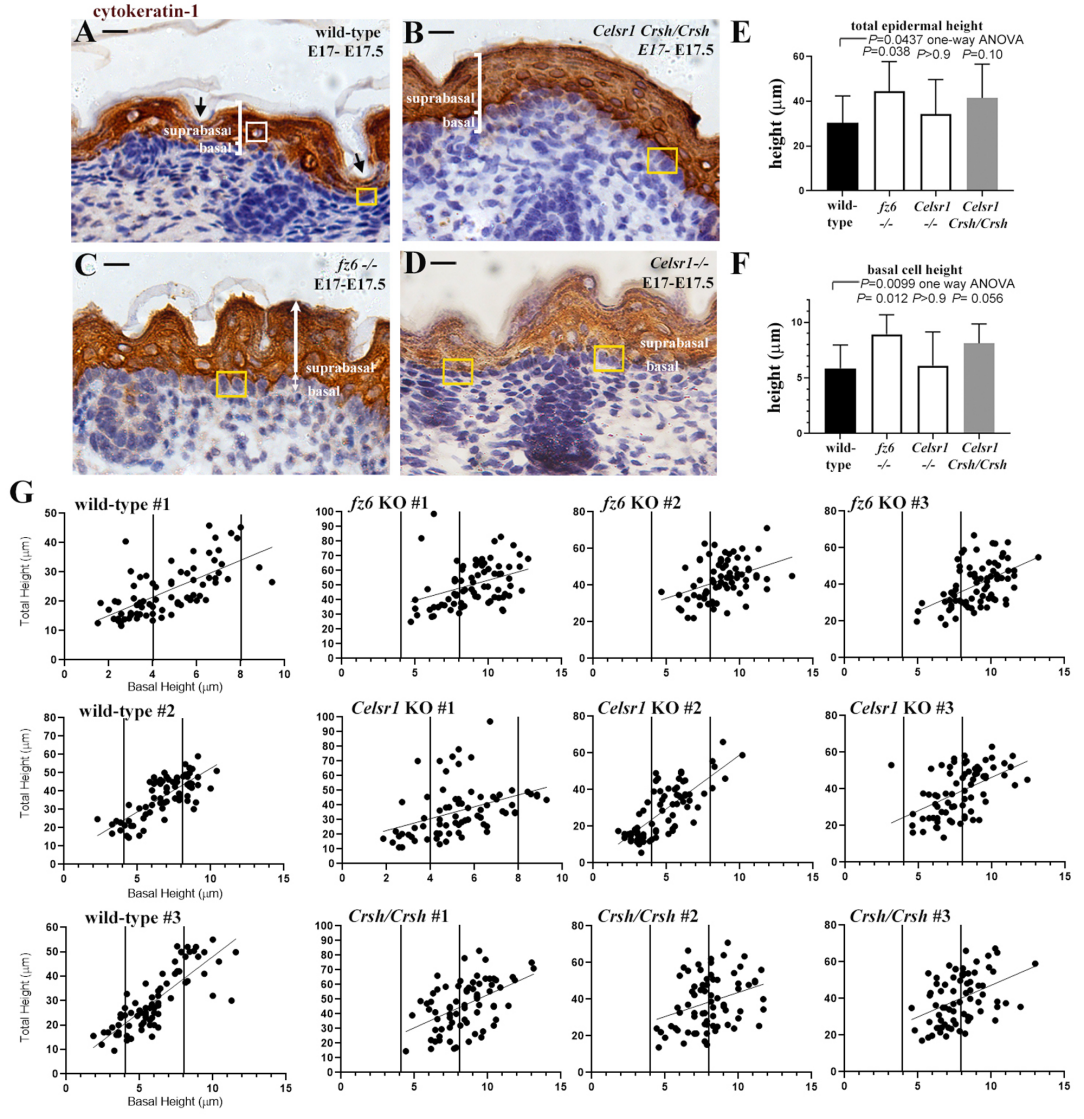
Planar Cell Polarity (PCP) pathway components expressed in the skin basal layer determine later stage basal cell height. ( **A**- **D**) Representative longitudinal wax sections of E17-E17.5 mid-trunk back skin from ( **A**) wild-type ( **B**) *Crash* homozygotes ( *Crsh/Crsh*), a mutant in Celsr1 ( Curtin *et al.,* 2003) which expresses Celsr1 protein but lacks PCP protein planar asymmetry, ( **C**) *frizzled6 (fz6*-/-; Wang *et al.,* 2010) and ( **D**) *Celsr1 (Celsr1*-/-; Ravni *et al.,* 2009) knock-out embryos. Skin sections were immunostained with cytokeratin-1 to visualise suprabasal cells, counterstain for nuclei is blue. White brackets label basal and suprabasal layers in ( **A**, **B**). Black arrows in ( **A**) label thinner sections of the suprabasal layer correlating with squamous basal and suprabasal cells. White box in ( **A**) labels a rounded nucleus of a suprabasal cell. Yellow box in ( **A**) highlights a squamous basal cell. Yellow boxes in ( **B**, **C**) label columnar basal cells. Yellow boxes in ( **D**) label squamous (left hand box) and cuboidal (right hand box) basal cells. White arrows in ( **C**) denote basal and suprabasal height measurements taken. Skin surface is to the top, scale bar is 20μm in all cases. ( **E**, **F**) Histograms of total epidermal depth ( **E**) and basal cell height ( **F**), n=75 measurements from wax sections from individual embryos, n=3 for each condition from at least two litters. Measurements made were taken every 10 basal cells across the inter-follicular epidermis. Measurements for wild-type are combined measurements for one wild-type littermate taken from one litter for each PCP mutant. Mean and SD are shown. Statistical analysis was one-way ANOVA with Bonferroni post-hoc test between mean values for n=3 biological replicates for each condition, individual values show comparisons between individual mutants and wild-type. ( **G**) Correlation plots of total epidermal height at location of individual basal cells against individual basal cell height for each embryo analysed. Measurements made are shown by white arrows in ( **C**) and were taken every 10 basal cells across the inter-follicular basal layer of mid-trunk back skin. 75 measurements were taken from n=3 sections from each of n=3 embryos (from at least two litters) for each condition.

A curious observation about E17-E17.5 *Crsh/Crsh* embryonic skin is that the suprabasal layer was thickened compared to wild-type, with visibly more rounded, cytokeratin-1 expressing suprabasal cells (nuclear shape was taken as a proxy for cell shape) overlying the basal layer in mutant skin compared to wild-type littermates ( Figure 1A, B, E) ( Formstone, 2022). *Crsh/Crsh* mutants exhibited an open neural tube ( Curtin *et al.*, 2003), which may contribute to the skin thickening phenotype ( Box *et al.*, 2019), however, study of *fz6* homozygote knockout (KO: -/-) mouse embryos, which exhibit closed neural tubes, revealed that the epidermis of *fz6-/-* embryos was significantly thickened compared to wild-type embryos ( Figure 1A,C,E), suggesting an underlying tissue intrinsic defect.

**Figure 2.**
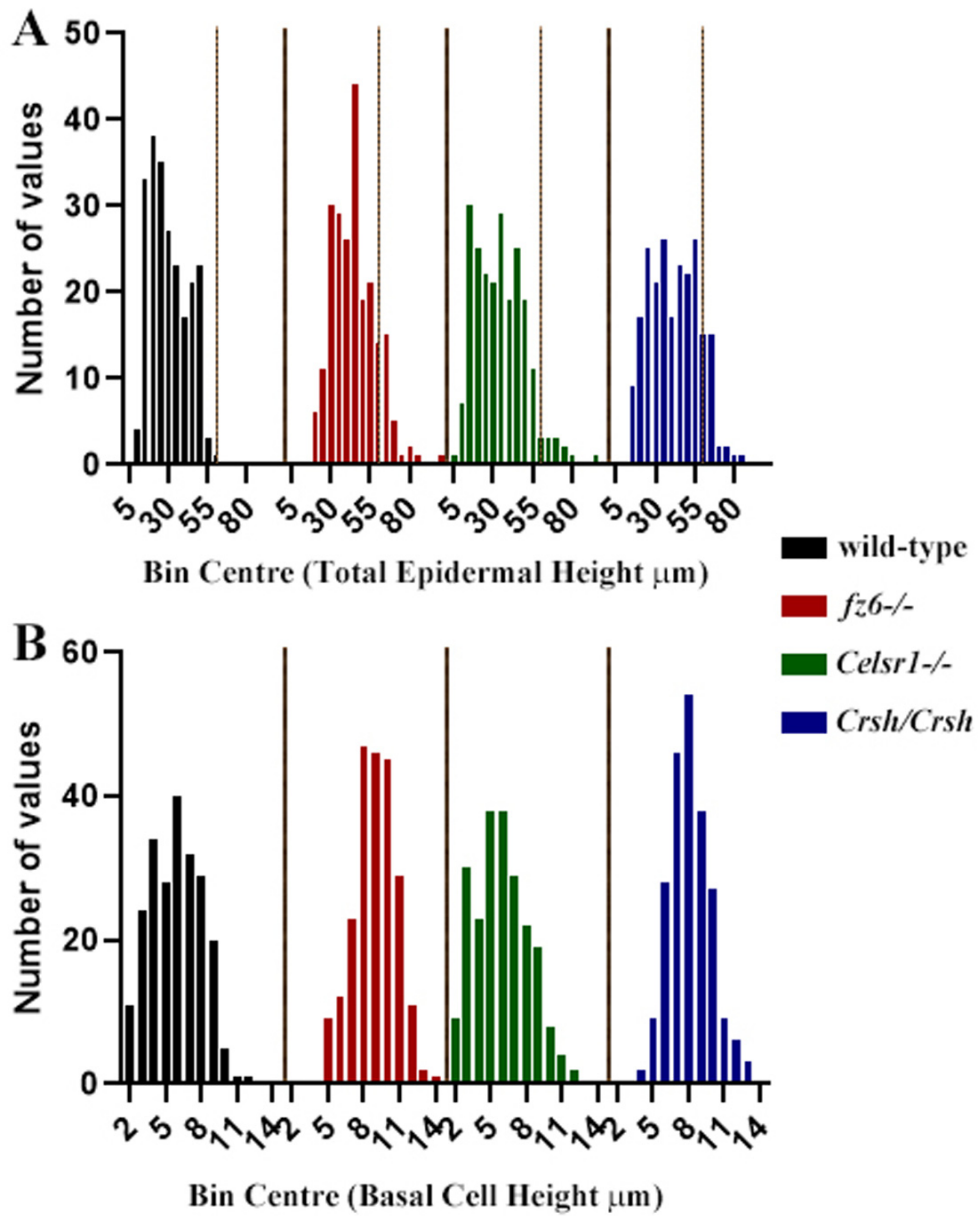
Each PCP mutant exhibits evidence of a thickened epidermis. Frequency plots for total epidermal ( **A**) and basal cell ( **B**) height made using GraphPad Prism. Prism allocated bin centres using the same measurements as for graphs and correlation plots shown in Figure 1.

Scatter plots of total epidermal height against basal cell height (total epidermal height was measured for each individual basal cell for which cell height was measured) were generated for individual embryos revealing strong similarities between *fz6-/-* and *Crsh/Crsh* embryos compared to wild-type ( Figure 1G). Individual *Celsr1-/-* embryos however demonstrated variability in phenotypes (representative data is shown; Figure 1G) which was consistent across more than three litters (embryos shown are representative for the different phenotypes observed in n=8 embryos analysed). More than one *Celsr1-/-* embryo exhibited evidence of thickened epidermis ( Figure 1G). Indeed, graphs showing the frequency distribution of total epidermal height revealed values greater than 59μm (shown by thin vertical line, Figure 2A) for all PCP mutants, which was the maximum height measured in wild-type embryos. Frequency data for *Celsr1-/-* embryos was generated using total and basal height measurements from each of the representative embryos shown in Figure 1G. Further study at earlier embryonic stages are required in this mutant to evaluate the underlying basis of the observed variable phenotype.

**Figure 3.**
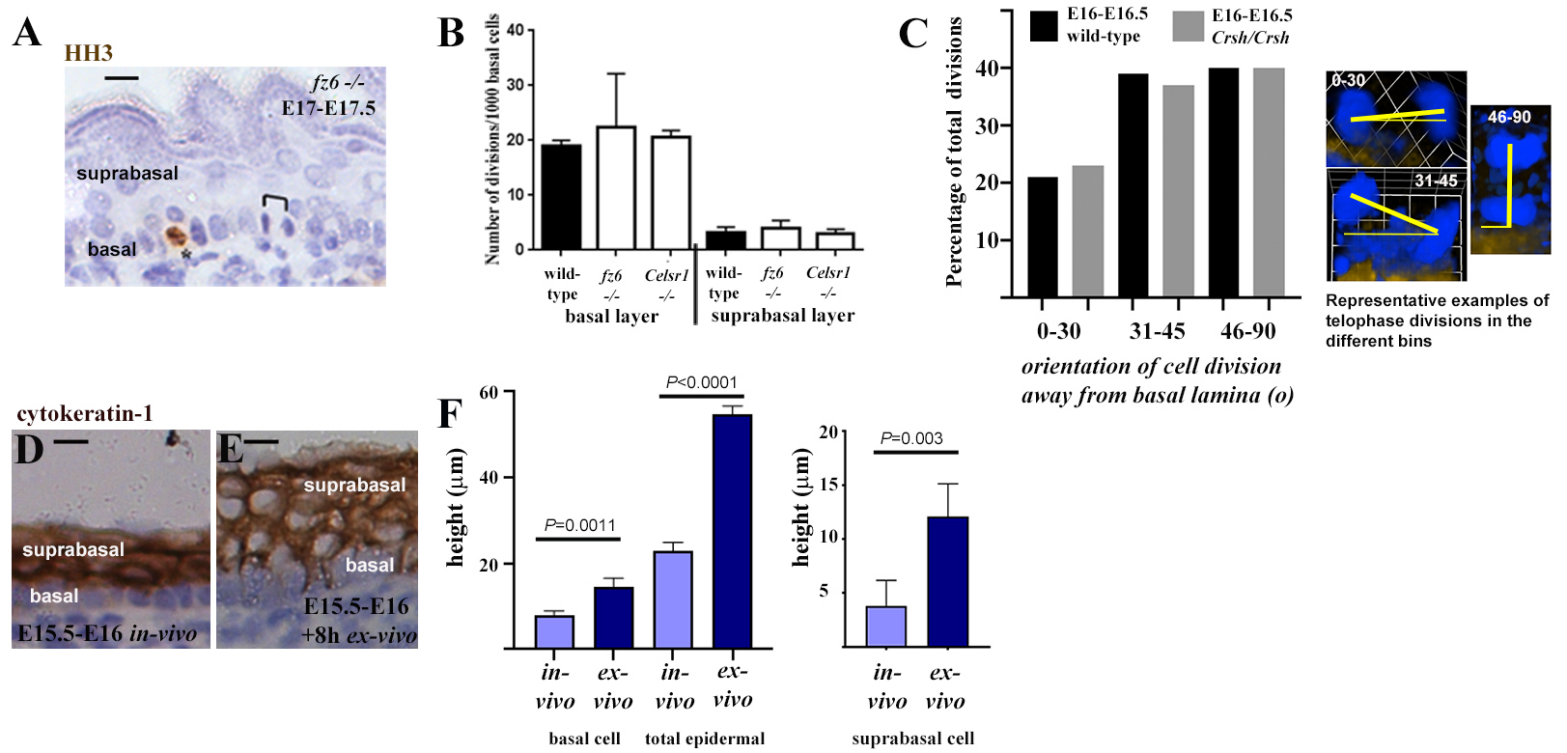
Cell proliferation index and orientation of cell division in PCP mutants are similar to wild-type. ( **A**) Representative longitudinal wax section of mid-trunk back skin, asterisk labels a dividing cell in the basal layer stained with pHH3 (brown stain), black bracket labels a telophase division. Scale bar is 10μm. ( **B**) Histogram showing number of cell divisions scored per 1000 basal cells in both basal and suprabasal layers of mid-trunk back skin from wild-type and PCP mouse mutants, n=3 embryos from 2 litters for each condition. Statistical analysis was Students t-test of biological replicates from wild-type and each mutant, no significant differences were found. ( **C**) Percentage of total divisions which exhibit specific angles of orientation away from the basal lamina. Only telophase divisions were scored within inter-follicular epidermis. Angles are binned 0–30, 30–45, 46–60, 61–75 and 76–90 to represent horizontal (0–30), oblique (30–45, 46–60) and vertical (61–75) divisions ( Lechler & Fuchs, 2005). Frozen longitudinal sections of mid-trunk back skin were analysed from n=3 wild-type and n=3 *Crsh/Crsh* skins from four litters in each case, a total of n=37 wild-type divisions and n=36 *Crsh/Crsh* divisions were scored. 3D images of Z-stacks for representative telophase divisions are shown. Nuclei are in blue, yellow lines label angle of cell division orientation relative to the basal lamina (gold staining). ( **D**, **E**) Representative image of wax sections of E15.5-E16 wild-type epidermis *in vivo* and explants of E15.5-E16 wild-type epidermis following an 8 hour (8h) suspension culture ( *ex-vivo*), n=3 whole embryos and n=3 explants, taken from the same two litters. Scale bar is 10μm. Counterstain of nuclei is in blue. ( **F**) Histograms of basal cell height, suprabasal cell height and total epidermal height at E15.5-E16 *in vivo* and E15.5-E16 + 8h in suspension culture ( *ex-vivo*). Mean and SD are shown, statistical analysis was Students t-test of biological replicates (n=3), n=15 measurements per biological replicate.

 No significant changes in cell proliferation index in either basal or suprabasal layers of *fz6*-/- embryos were observed that might account for the epidermal thickening phenotype ( Figure 3A,B). From E15.5 onwards, Inscuteable and LGN protein crescents on the apical side of epidermal basal cells are reported to define oblique and vertical epidermal basal cell divisions as asymmetric, giving rise to a basal cell daughter as well as a differentiating suprabasal cell daughter ( Lechler & Fuchs, 2005; Williams *et al.*, 2011). Further support for this model was provided recently by Damen *et al.* (2021). The proportion of basal cell divisions exhibiting horizontal, oblique and vertical orientations in E16-E16.5 *Crsh/Crsh* skin compared to wild-type was similar ( Figure 3C), consistent with a recent study of *fz6*-/- skin (see supplementary data, Box *et al.*, 2019). Thus, the ratio of symmetric to asymmetric divisions, which promote epidermal stratification in later stage skin, appears to be undisturbed in *fz6* and *Celsr1 Crsh/Crsh* PCP mutants.

A frequency distribution of values for basal cell height measurements from images of wax sections immunostained with cytokeratin-1, which labels suprabasal cells, demonstrated that the skin basal layer (nuclei of which were co-stained blue) in wild-type contained a mixture of tall (columnar), square (cuboidal) and flattened (squamous) epithelial cells with a bias towards squamous-type: most frequent height was found for bin centre 6μm ( Figure 2B) Conversely, *fz6*-/- basal cells were predominantly columnar (most frequent heights were around 8-10μm; Figure 2B) whilst *Crsh/Crsh* basal cells exhibited bias towards columnar or cuboidal (most frequent height was found for bin centre 8μm; Figure 2B). Both *fz6*-/- and *Crsh/Crsh* basal cells were rarely less than 5μm in height ( Figure 2B).

Notably, the bias towards taller basal cells in *fz6*-/- and *Crsh/Crsh* correlated with suprabasal cells which were more visibly rounded in shape ( Figure 1B,C). In wild-type, squamous basal cells were generally overlain by a flattened suprabasal layer where suprabasal cells were squamous in appearance (black arrows, Figure 1A). Cuboidal/columnar wild-type basal cells however were overlain by a thicker suprabasal layer containing suprabasal cells that were rounded in appearance (white boxed area, Figure 1A). Correlation analysis on scatter plots where basal cell height was plotted against total epidermal height for wild-type embryos ( Figure 1G) revealed mean r-squared values for wild-types as +0.56 ( *P* value <0.0001) supporting a positive correlation between basal cell height and total epidermal height ( Figure 1G). Mean r-squared values for *fz6-/-* and *Crsh/Crsh* however were significantly lower 0.18 (r squared *P* value range of 0.0002- <0.0001) and 0.17 (r squared *P* value range of 0.0031- <0.0001) respectively. Mean r-squared value for representative *Celsr1-/-* embryos was 0.33 (r squared *P* value range of 0.0002- <0.0001). One way ANOVA (Bonferonni post-hoc test) across all PCP mutants and wild-type controls gave a *P* value of 0.024 with multiple comparisons revealing *P* values of 0.0266 ( *fz6*KO), 0.0223 ( *Crsh/Crsh*) and 0.22 ( *Celsr1-/-*) for individual mutants compared to wild-type controls. We found therefore a generally lower correlation between basal cell height and total epidermal height for all PCP mutants analysed.

Wax sections for *fz6-/-* and *Crsh/Crsh* mutants show visibly more rounded suprabasal cells, we were not able to robustly quantify suprabasal cell shape however in wax sections. One simple explanation for the presence of more rounded suprabasal cells directly above the more columnar height of basal cells observed in *fz6-/-* and *Crsh/Crsh* skin is direct mechanical contact. We have recently used suspension explant ( *ex-vivo*) culture to investigate skin morphogenesis and found that explants of wild-type surface ectoderm deform when excised away from the intact embryo, demonstrating that the embryonic skin is under extrinsic mechanical tension (Lumox culture: Panousopoulou *et al.*, 2016). Explant of E15.5-E16 mid-flank wild-type skin (+8h, Lumox suspension culture) also resulted in epidermal deformation Figure 3D,E). Basal cells became significantly taller and suprabasal cells became more rounded *ex-vivo* compared to *in vivo,* leading to a significant increase in epidermal depth ( Figure 3F). These data reveal that both basal and suprabasal cells respond to loss of extrinsic force by changing their height, indicating that coordination of tissue mechanics between epidermal layers could result in a thicker epidermis in PCP mutant embryos which exhibit a bias towards more columnar basal cells.

## Discussion

 Previous studies reveal a role for core-PCP proteins in determining epithelial cell shape ( Nishimura *et al.*, 2012; Shi *et al.*, 2014). Here it is reported that two different mouse mutants in core-PCP genes exhibit loss of squamous basal cells at later stages of embryonic epidermal development. Both *fz6* and *Celsr1 Crsh/Crsh* mutants exhibit a bias towards taller (more columnar or cuboidal) epidermal basal cells. A similar later stage thickening of the epidermis has also been reported in the *Vangl2 Lp/Lp* mutant, which was linked to the presence of taller basal cells and an increased frequency of oblique/perpendicular basal cell divisions at the expense of planar orientations much earlier in skin development i.e., at around E14.5 ( Box *et al.*, 2019). Damen *et al.* (2021) find that centriole loss in skin where p53-mediated cell death has been suppressed does not disrupt skin morphogenesis prior to E15.5. The authors proposed therefore a two-stage system (early/late) for epidermal development, with later stages (E15.5 onwards) relying on asymmetric basal cell divisions to supply new suprabasal cells Damen *et al.* (2021). In light of this model and the highly similar later stage epidermal thickening phenotype in *Crsh/Crsh* and *fz6-/-* mid-flank skins at E17-E17.5, it is proposed here that both *Crsh* homozygotes and *fz6-/-* exhibit a similar later stage tissue intrinsic defect in the generation of squamous basal cell shape.

R-squared values from scatter plots suggest that flattened (squamous) basal cell shape in later stage skin impacts on suprabasal cell shape. Suspension explants of E15.5-E16 wild-type skin provides evidence for a mechanical coordination in cell height between different epidermal layers. Significantly lower r-squared values for *fz6-/-* and *Crsh/Crsh* scatter plots compared to wild-type revealed however a low correlation between basal cell height and total epidermal height. This might be accounted for by there being a limit to the maximum height of individual suprabasal cells in the intact embryo. Given however that *fz3* becomes restricted to epidermal suprabasal layers at E17 ( Hung *et al.*, 2001) and *fz3*-/- E17 skin is also thickened ( Dong *et al.*, 2018, supplementary data) it is also entirely possible that PCP protein signalling between different epidermal layers also plays a role. Indeed, this may explain the variability in phenotype in *Celsr1-/-* mutants.

 In wild-type, squamous basal cells make up just 30% of the total number of basal cells in the later stage epidermis. Thus, PCP proteins appear to act locally within specific cell communities to generate squamous basal cells. Local PCP protein signalling is well-documented in the skin e.g., hair placodes use local planar oriented cell-cell rearrangements within the basal layer of the hair placode to create a cellular asymmetry, which couples to the global alignment of their subsequent oblique oriented down-growth into the underlying dermis ( Cetera *et al.*, 2018; Devenport & Fuchs, 2008). *Crash* and *Lp* homozygote hair placode/follicle phenotypes ( Devenport & Fuchs, 2008) also infer local disruption to PCP signalling. Additionally, E16 basal cells are reported to rely upon local cues from neighbouring interphase cells to orient their planar axis of cell division ( Oozeer *et al.*, 2017). PCP signalling processes in the skin are defined by the asymmetric enrichment of PCP proteins along the axis of planar polarity ( Devenport & Fuchs, 2008). It is not certain, however, whether asymmetric PCP protein distribution is necessary to generate squamous basal cell shape in later stage skin. Fz6, for example, is not unique in the Frizzled family in playing a role in epithelial cell height. Cell height is reduced in the monolayer epithelium of developing mouse lung tubules of *fz2-/-* embryos ( Kadzik *et al.*, 2014).

In conclusion, we find that the presence of squamous basal cells in the later stage mouse embryonic epidermis is dependent upon core-PCP protein function. Further study is necessary to understand whether squamous versus columnar basal cell height impacts overlying suprabasal cell shape and whether PCP proteins impact on their potential association. The findings presented here also support a role for core-PCP proteins in the ‘outside-in’ (radial) organisation of the developing mammalian skin. Future work will focus on a more detailed analysis of the correlation between basal and suprabasal cell shape in wholemounted skin at earlier embryonic stages which will enable packing and cell shape in both layers to be fully investigated.

## Data Availability

OSF: Formstone 2022 WT Open Research. https://doi.org/10.17605/OSF.IO/G39WT ( Formstone, 2022) This project contains the following underlying data: Data files for Tables Figure 1 file Data for epidermal height and orientation of cell division – E17-E17.5 epidermis Data for orientation of cell division Data for epidermal height – E15.5-E16 explants Photomicrographs for Formstone 2022 Wellcome Trust Open Research Photomicrographs for wild-type epidermis Photomicrographs for Celsr1 KO epidermis Photomicrographs for fz6 KO epidermis Photomicrographs for Celsr1 Cras/Crash epidermis Photomicrographs for E15.5-E16 whole embryo and explants OSF: ARRIVE checklist for ‘Planar Cell Polarity protein-dependent basal cell height in the later stage mouse epidermis impacts on the shape of overlying suprabasal cells’. https://osf.io/ts8fr/?view_only=16c87f5bf6ba4318a12d9a8c3bfde25a ( Formstone, 2022) Data are available under the terms of the Creative Commons Zero "No rights reserved" data waiver (CC0 1.0 Public domain dedication).
