# Supplementary material for: Planar cell polarity proteins determine basal cell height in the later stage embryonic mouse epidermis'
Source: Wellcome Open Res. 2023 Jan 19;7:138. Originally published 2022 Apr 19. [Version 2] doi: 10.12688/wellcomeopenres.17733.2 (PMC10020738; doi:10.12688/wellcomeopenres.17733.2)
Supplement: Peer review history

# Peer review: Reviewer response for version 2

**DOI:** 10.21956/wellcomeopenres.20902.r54329

**Published:** 2023-03-16

**Authors:** David Strutt, Helen Strutt

**Affiliations:** 1 School of Biosciences, University of Sheffield, Sheffield, UK; 2 The University of Sheffield, Sheffield, UK

The authors have taken on board our suggestions and we have no further comments.

Is the work clearly and accurately presented and does it cite the current literature?

Yes

If applicable, is the statistical analysis and its interpretation appropriate?

Partly

Are all the source data underlying the results available to ensure full reproducibility?

Yes

Is the study design appropriate and is the work technically sound?

Yes

Are the conclusions drawn adequately supported by the results?

Yes

Are sufficient details of methods and analysis provided to allow replication by others?

Yes

Reviewer Expertise:

Cell and developmental biology of planar polarity

We confirm that we have read this submission and believe that we have an appropriate level of expertise to confirm that it is of an acceptable scientific standard.

# Peer review: Reviewer response for version 2

**DOI:** 10.21956/wellcomeopenres.20902.r54328

**Published:** 2023-01-26

**Authors:** Hisham Bazzi

**Affiliations:** 1 Department of Dermatology and Venereology, CECAD Excellence Cluster, Medical Faculty, University of Cologne, Cologne, Germany

No further comments.

Is the work clearly and accurately presented and does it cite the current literature?

Yes

If applicable, is the statistical analysis and its interpretation appropriate?

Yes

Are all the source data underlying the results available to ensure full reproducibility?

Yes

Is the study design appropriate and is the work technically sound?

Yes

Are the conclusions drawn adequately supported by the results?

Partly

Are sufficient details of methods and analysis provided to allow replication by others?

Yes

Reviewer Expertise:

NA

I confirm that I have read this submission and believe that I have an appropriate level of expertise to confirm that it is of an acceptable scientific standard.

# Peer review: Reviewer response for version 1

**Published:** 2022-07-26

**Authors:** David Strutt, Helen Strutt

**Affiliations:** 1 School of Biosciences, University of Sheffield, Sheffield, UK; 2 The University of Sheffield, Sheffield, UK

This short manuscript follows up on previous observations of epidermal thickening in Vangl2 mutant mice. The authors report a similar thickening of the epidermis in Celsr1 and Fz6 mutant animals, and describe cell shape changes in both the basal cell layer and the suprabasal cell layer.

1. A diagram of the epidermal layers would be useful to help orient less expert readers. An alternative to a cartoon might be clear ‘brackets’ beside one of the figure panels indicating the cell layers. The white arrows in panel C partly address this point, but are rather thin and hard to see.
  Could the authors zoom in on one of the panels B-D and point out the basal cell layer, and also examples of squamous, cuboidal and columnar basal cells?
2. Panel E, F: Total epidermal depth and basal cell height are measured, n = 75 measurements from 3 embryos, measurements taken every 10 basal cells. Given the very low p-values, we wonder if the ‘n’ value used for the statistics was in fact n=75 rather than n=3. In our view, the true n is 3 (number of embryos), not 75. The 25 measurements within an embryo seem to fall into the category of technical replicates, not biological replicates. We would suggest taking an average per embryo and doing stats between embryos (if this wasn’t what was done). Most likely the result will still be significant (or close to significant), but in either case reporting the actual p-values would provide useful information.
  Regarding the statistical test used, the legend indicates ‘Students t-test’, however multiple comparisons are shown and the Methods mention both t-tests and ANOVA. We think the appropriate test for multiple comparisons is ANOVA with post-hoc correction for multiple samples (e.g. Bonferroni – we think ‘Bonferretti’ in the Methods is a typo?). Could the authors clarify the test used?
  Panel H legend also states t-test, but as there are 3 genotypes, ANOVA looks more appropriate. Although the authors state in the text that differences are not significant, giving the actual p-value would be more informative (e.g. p = 0.051 is conventionally regarded as ‘not significant’ while p =0.049 is regarded as ‘significant’, but both p-values are very similar and it’s hard to justify regarding them as qualitatively different).
  Panel M: We have similar questions regarding technical vs biological replicates and the appropriate n number.
3. It would be nice (but not essential) to have a sample image to accompany panel I, showing examples of different orientations of cell division.
4. Panel J: Celsr, Fz6 and Crsh mutants had more columnar and cuboidal cells relative to wild-type. The total epidermal depth for each of these classes was then plotted, with the conclusion that having columnar/cuboidal cells rather than flat/squamous cells correlates with overall epidermal thickness.
  As there will be a range of cell heights within each class, we wonder whether it might be also useful to show scatter plots of individual basal cell height versus total epidermal thickness? This might firm up the connection between basal cell height and epidermal thickness.
  We were again uncertain about the stats. Is the p-value given the overall ANOVA p-value for comparisons of epidermal depth for all classes, or for comparison of epidermal depth in columnar vs squamous (as the bars above the graphs might imply)? Clarifying this in the figure legend would be good. If the former, why not also show the pair-wise values on the graph?
  There is also the question of the correct n number for the ANOVA. Averaging within animals and then comparing between animals would again seem most appropriate? However, there may not be enough measurements in each animal for some classes (e.g. squamous basal cells) to do this. This is why scatter plots of individual cell height vs epidermal depth might be more valid, using either pooled data or plotting each animal individually, and looking at the r-squared values for these plots to show a correlation.
5. The authors say the bias towards taller basal cells in fz6-/-, Celsr1-/- and Crsh/Crsh correlated with suprabasal cells which were more rounded in shape. While not essential, can this be quantitated in some way?

Is the work clearly and accurately presented and does it cite the current literature?

Yes

If applicable, is the statistical analysis and its interpretation appropriate?

Partly

Are all the source data underlying the results available to ensure full reproducibility?

Yes

Is the study design appropriate and is the work technically sound?

Yes

Are the conclusions drawn adequately supported by the results?

Yes

Are sufficient details of methods and analysis provided to allow replication by others?

Yes

Reviewer Expertise:

Cell and developmental biology of planar polarity

We confirm that we have read this submission and believe that we have an appropriate level of expertise to confirm that it is of an acceptable scientific standard, however we have significant reservations, as outlined above.

# Peer review: Reviewer response for version 1

**DOI:** 10.21956/wellcomeopenres.19624.r50043

**Published:** 2022-05-13

**Authors:** Hisham Bazzi

**Affiliations:** 1 Department of Dermatology and Venereology, CECAD Excellence Cluster, Medical Faculty, University of Cologne, Cologne, Germany

The manuscript by Hobbs and Formstone, “Planar cell polarity protein-dependent basal cell height in the later stage of embryonic mouse epidermis impacts on the shape of overlying suprabasal cells” reports a brief and targeted analyses of epidermal dynamics in the planar cell polarity (PCP) constitutive mutants for Fzd6 and Celsr1. The authors focus mainly on embryonic day (E) 17.5 of skin development and the data correlate epidermal thickness with basal cell height as well as suprabasal cell shape.

 The data in the manuscript extend previous findings by Box *et al*, 2019 on Vangl2 mutants to other PCP mutants, namely Celsr1 and Fzd6. The authors conclude that “basal cell height…impacts on the shape of overlying suprabasal cells”; however, a causal relationship is difficult to establish from correlation in fixed samples and at a single timepoint during development. The authors acknowledge the correlation in the abstract and main text but the title asserts a causal link with the word “impacts on”. A more objective conclusion from the data maybe that the thickness of the epidermis correlates with the packing of the cells and their shapes in both the basal and suprabasal layers, which also applies to the data on the ex-vivo skin cultures in K-M, and perhaps the basal cell packing reported in Box et al., 2019. In my opinion, the outstanding questions are: why is the cellular packing different in PCP mutants? If it does not correlate with proliferation rates as shown in Fig. 1H (and Box *et al*., 2019), then what about cellular densities and cell sizes in both the basal and suprabasal layers from E13.5 – E17.5? A more thorough analysis of PCP mutants is required to address these questions.

 The authors raise an interesting point on a potential non-cell autonomous role for PCP proteins that are expressed in the basal layer and affect the suprabasal layers, which is an area for further investigation into the relationships between the different layers of the epidermis.

 Below are more specific comments:

1. Fig. 1A: The examples of squamous suprabasal cells shown by the black arrows coincide with the troughs of epidermal ridges, which are hallmarks of the morphogenetic processes that occur in the skin after E15.5. The suprabasal cell shape was not quantified to support the author’s conclusion regarding the correlation with the basal cell height.
2. Fig. 1D: The orientation of the section shown appears slanted and may impact on the thickness of the epidermis and cell shapes.
3. Damen *et al.* 2021 is not correctly cited in reference to the roles of asymmetric cell division in the later stages of epidermal development. The author’s conclusion in this article is more focused on the roles of delamination than asymmetric cell division per se.
4. Perhaps a typo in reference to Box *et al*. 2019 analyzing Fzd6 mutants, when the authors likely mean Vangl2 mutants instead.

Is the work clearly and accurately presented and does it cite the current literature?

Yes

If applicable, is the statistical analysis and its interpretation appropriate?

Yes

Are all the source data underlying the results available to ensure full reproducibility?

Yes

Is the study design appropriate and is the work technically sound?

Yes

Are the conclusions drawn adequately supported by the results?

Partly

Are sufficient details of methods and analysis provided to allow replication by others?

Yes

Reviewer Expertise:

NA

I confirm that I have read this submission and believe that I have an appropriate level of expertise to confirm that it is of an acceptable scientific standard, however I have significant reservations, as outlined above.
